# Peritoneal tuberculosis in pregnancy: a case report

**Published:** 2012-07-05

**Authors:** Fatima Zohra Fdili Alaoui, Myriem Rachad, Hikmat Chaara, Hakima Bouguern, Moulay Abdilah Melhouf

**Affiliations:** 1 Department of gynecology and obstetrics II, CHU Hassan II, Fez, Morocco

**Keywords:** Peritoneal tuberculosis, pregnancy, diagnosis

## Abstract

Peritoneal tuberculosis in pregnancy is one of the least common forms of extrapulmonory tuberculosis in pregnancy. Early diagnosis is important to prevent obstetrical and neonatal morbidity. We report the case of a 37-year-old pregnant woman who presented with abdominal volume increase, night-sweat, anorexia, loss of weight and abdominal pain at 23 weeks. A peritoneal laparoscopic biopsy was performed and confirmed the diagnosis of tuberculous peritonitis. The patient received antituberculosis chemotherapy. The recovery was good as gave birth to a healthy infant of 3200Kg at 37th week's gestation by vaginal delivery.

## Introduction

Peritoneal tuberculosis in pregnancy is one of the least common forms of extrapulmonory tuberculosis in pregnancy. Early diagnosis is important to prevent obstetrical and neonatal morbidity. We present the case of a pregnant woman with peritoneal tuberculosis. The diagnostic and therapeutic problems are discussed, and the relevant literature is briefly reviewed. The objective of our work is to help clinicians to diagnose and treat these patients early.

## Patient and case report

A 37-year old woman, multiparous, of low socioeconomic status, was referred to our department at 23 weeks gestation for ascites. The patient reported a notion of abdominal volume increase one month ago, with night-sweat, anorexia, weight loss and abdominal pain which increases in intensity for subsequent weeks. Clinical examination revealed no fever, weight at 52Kg and height 1m60, (BMI= 20, 3), her abdomen was distended, diffused dullness on percussion, without pathological masses.

**Figure 1:**
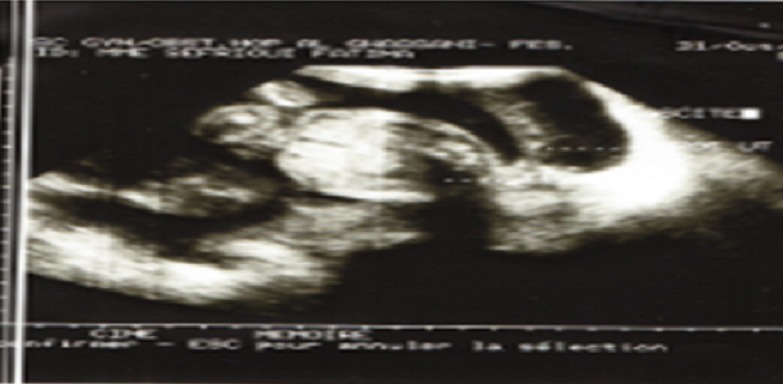
Obstetric ultrasound image showing a pregnancy at 23 weeks of amenorrhoea with ascites

**Figure 2:**
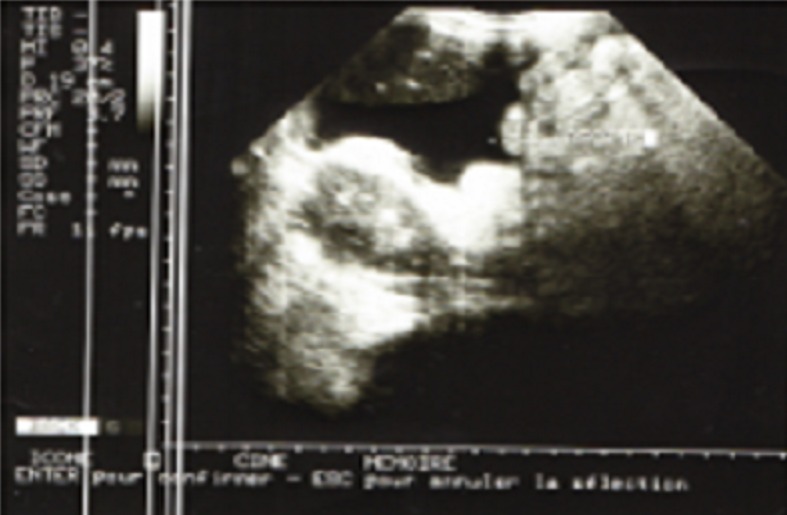
Obstetric ultrasound image showing a pregnancy at 23 weeks of amenorrhoea with ascites filling the interhepato-diaphragmatic space

Laboratory investigation showed mild normochrom and normocytic anemia (Hemoglobin level 10,6g/dl) without leucocytosis. Abdominal ultrasound (Figure 1, Figure 2) showed intra-abdominal fluid, mainly in the lowest abdomen, and a thickened peritoneum. No ovarian mass was indentified on ultrasound. A chest X –ray showed no active lesion or old lesion compatible with pulmonary tuberculosis. A tuberculin skin test was positive. Acid-fast bacilli in sputum were not seen on sputum microscopy (Ziehl Nielson stain).

A peritoneal laparoscopic biopsy was performed to confirm the diagnosis. About 2.5 liter of straw-colored ascitis fluid was removed from the abdomen, there were multiple extensive nodules with central necrosis on the peritoneal surface and milary deposits on the intestine. Excision biopsy was taken from the peritoneal lesions. The final histologic report confirmed chronic granulomas with caseous necrosis and multinucleated giant cells. The patient received antituberculous chemotherapy regimen with rifampicin (R), isoniazid (H), pyrazinamide (Z) for two months and 4 months of RH.

The general condition of the patient improved significantly and she was discharged from the hospital on postoperative day 15. The pregnancy continued without any problem, prenatal ultrasonographic findings showed a single fetus in a cephalic presentation and biometric measurements were consistent with the date of pregnancy. The patient gave birth to a healthy infant of 3200Kg at 37th week's gestation by vaginal delivery.

## Discussion

Tuberculosis (TB) in pregnant women has been a concern since the days of Hippocrates. Because of improved living conditions and discovery of effective chemotherapy, the incidence of TB has rapidly declined. However, recently there has been resurgence of the disease due most likely to many factors, including the human immunodeficiency virus (HIV) epidemic, drug abuse, poverty, homelessness, deterioration in the health care infrastructure, and increasing number of cases among immigrants. So, tuberculosis is still a health problem in developing countries.

In pregnancy, the incidence of tuberculosis is low; moreover, peritoneal tuberculosis (PT) in pregnancy is uncommon because infertility is the commonest sign if tuberculosis involves the genitals and/or peritoneum [1].

PT typically involves the entire abdominal cavity (omentum, intestinal tract, liver, spleen and female genital tract), in addition to the parietal and visceral peritoneum. It represents approximately 12% of all tuberculosis cases and is occasionally seen in association with the pulmonary or the disseminated form of the disease. A possible mechanism to explain the pathogenesis of PT is the reactivation of latent tuberculous foci in the peritoneum or hematogenous spread from primary pulmonary tuberculosis. However, the primary focus in the lungs is often healed completely, thereby precluding its identification, despite careful examination of the patient, as occurred in our case [2].

The clinical manifestations of tuberculous peritonitis progress insidiously. Fever, chills, weight loss and abdominal pain are common complaints. Most patients require extensive diagnosis work-up because of unexplained prolonged febrile illness, ascites and elevated CA 125 level. PT is often misdiagnosed as advanced ovarian cancer [3–5].

Ascites is present in almost all patients; the fluid is exsudative (protein >2.5g/dl) with predomination of mononuclear cells; however, 10% of patients may have an initial neutrophilic response. Bacteriologic examination of the ascitic fluid is not always diagnostic: Acid-fast smears are rarely positive in tuberculous peritonitis, and conventional cultures yield the pathogen in only 25% of cases [2].

Elevation of the serum CA125 titer in pregnancy is not pathognomonic because elevated serum level of CA125 can be elevated even in benign diseases including peritonitis. However, the levels of CA125 have been less than 500 U/ml, and it could be used as a follow-up marker in patients treated for peritoneal tuberculosis [6, 7]. The presence of adenosine deaminase activity is a useful test in the diagnosis: the levels above 33U/l are 100% sensitive and 95% specific to the diagnosis.

The sensitivity of computed tomography (CT) scan in the prediction of tuberculosis is 69%. Patients with tuberculosis were likely to show mesenteric changes, macronodules (>5mm in diameter), splenomegaly, and splenic calcification on CT imaging [1].

Accurate diagnosis requires histopathological examination following image-guided biopsy (when possible), exploratory laparotomy or diagnostic laparoscopy. Bacteriologic examination of the biopsy specimen should be performed, because this could be positive for tuberculosis when histological examination is negative. This examination includes the identification of acid-fast bacilli (Ziehl-Neelsen staining positive), positive culture for Mycobacterium tuberculosis and positive PCR for M tuberculosis complex [8]. Early diagnosis and treatment of peritoneal tuberculosis in pregnancy are important in minimizing adverse obstetrical and neonatal effects [9, 10].

## Conclusion

Peritoneal tuberculosis should be suspected in ascites during pregnancy. The clinical manifestations progress insidiously. It is difficult to identify peritoneal tuberculosis in pregnancy by radiologic evaluation and laboratory. The diagnosis can be confused with ovarian cancer especially in front of the presence of the ascite. Accurate diagnosis requires histopathological examination following image-guided biopsy (when possible), exploratory laparotomy or diagnostic laparoscopy. Bacteriologic examination of the biopsy specimen should be performed because this could be positive for tuberculosis when histological examination is negative. Early diagnosis is important to prevent obstetrical and neonatal morbidity
